# Dupilumab may be an alternative option in the treatment of acquired reactive perforating collagenosis combined with AD

**DOI:** 10.1002/iid3.574

**Published:** 2021-12-24

**Authors:** Yang Ying, Chen Shuang, Zhang Zhen‐Ying

**Affiliations:** ^1^ Department of Dermatology The University of Hong Kong‐Shenzhen Hospital Shenzhen Guangdong China; ^2^ Department of Pathology The University of Hong Kong‐Shenzhen Hospital Shenzhen Guangdong China

**Keywords:** acquired reactive perforating collagenosis, atopic dermatitis, dupilumab

## Abstract

The management of acquired reactive perforating collagenosis (ARPC) is challenging. Here, we shared two cases of ARPC combined with elderly atopic dermatitis (AD) that did not respond well to conventional treatment but responded well to the monotherapy of dupilumab, which suggests that dupilumab may be an alternative option for the treatment.

## Graphical abstract

The management of acquired reactive perforating collagenosis (ARPC) is challenging. Our successful experience in ARPC suggests that dupilumab is a new choice for the treatment.

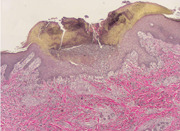

## 1 INTRODUCTION

Acquired reactive perforating collagenosis (ARPC) is one of the groups of acquired perforating dermatosis (APD), other APD diseases include acquired perforating folliculitis, and acquired elastosis perforans serpiginosa.^1^ The diagnostic criteria of ARPC require all of the following criteria to be met: (1) histopathological findings of elimination of necrotic basophilic collagen tissue into a cup‐shaped epidermal depression, (2) clinical presentation of umbilicated papules or nodules with a central adherent keratotic plug, and (3) onset of skin lesions after the age of 18 years.^2^ Moreover, pruritus is present and can be severe. The pathogenesis and etiology of ARPC are still unknown. Diabetes and chronic kidney disease are considered to be comorbidities of ARPC.^2^

The management of ARPC can be challenging. Antihistamines, topical steroids, and emollients are the most commonly used therapies. Systemic steroids, intralesional steroids and doxycycline, acitretin, allopurinol, and narrowband UVB (NB‐UVB), psoralen plus UVA therapy have also been used for the management of ARPC.^1^

Dupilumab is a fully human monoclonal antibody against interleukin‐4 receptor α (IL‐4Rα), which has been proved to have a good effect on pruritic disorders. Here, we would like to share two cases of ARPC combined with elderly atopic dermatitis (AD) that did not respond well to conventional treatment but responded well to the monotherapy of dupilumab.

## 2 CASE REPORTS

### 2.1 Patient 1

**Figure 1:**
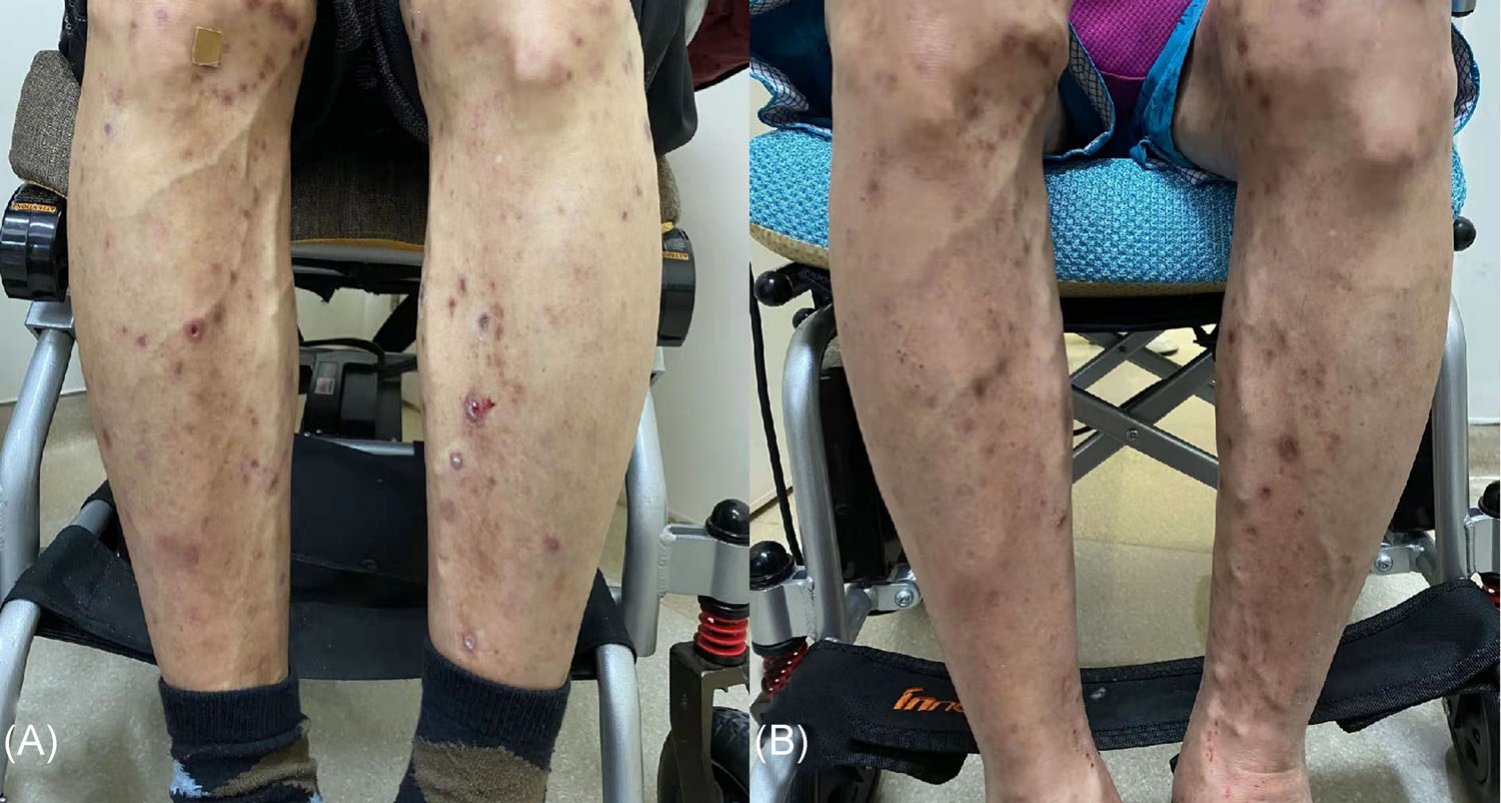
Clinical picture of Patient 1 before (A) and after (B) 3 months of treatment with dupilumab

**Figure 2:**
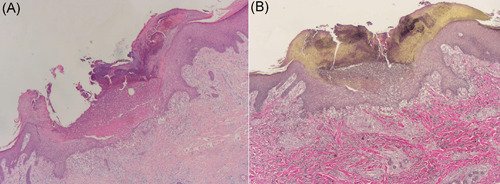
Histopathological picture (A) and Verhoeff‐van Gieson staining (B) for Patient 1

A 71‐year‐old man complained of relapsing pruritus papules and nodules on his trunk and limbs for 5 years. He showed poor response to oral antihistamines and topical steroids. One year ago, his lesions subsided and his itching sense eased after NB‐UVB phototherapy treatment for 12 weeks. Three months later, lesions with severe itches reoccurred and cannot be controlled even after rephototherapy combined by topical corticosteroids wet wrap. His medical history revealed essential hypertension, coronary heart disease, stroke, interstitial pneumonia, and type 2 diabetes for longer periods of years to decades. Scattered dry erythema, scratches, umbilicated papules, or nodules with symmetric distribution were noted on his chest, abdomen, and especially extremities (Figure 1A). Histopathological examination for umbilicated papule showed a cup‐shaped depression in the epidermis, necrotic and degenerated collagen and white blood cell debris were seen below, and necrotic collagen was seen to penetrate the epidermis (Figure 2A). Verhoeff‐van Gieson staining showed no elastic fibers but collagen fibers penetrated the epidermis (Figure 2B). Laboratory examination revealed the increased eosinophils count (1.53 × 10^9^/L), obvious increased level of total immunoglobulin E (IgE) (12,092 IU/ml), mild increased fasting plasma glucose (6.9 mmol/L), and mild increased serum creatinine (121 μmol/L). The diagnosis of ARPC and elderly AD was made. After excluding relevant contraindications, the patient was treated with monotherapy of dupilumab in a routine way. Three months later, obvious relief of skin lesions and pruritus was obtained (Figure 1B), and the patient did not report any adverse effects. During the follow‐up period, the patient continued to use dupilumab without recurrence.

#### 2.1.1 Patient 2

**Figure 3:**
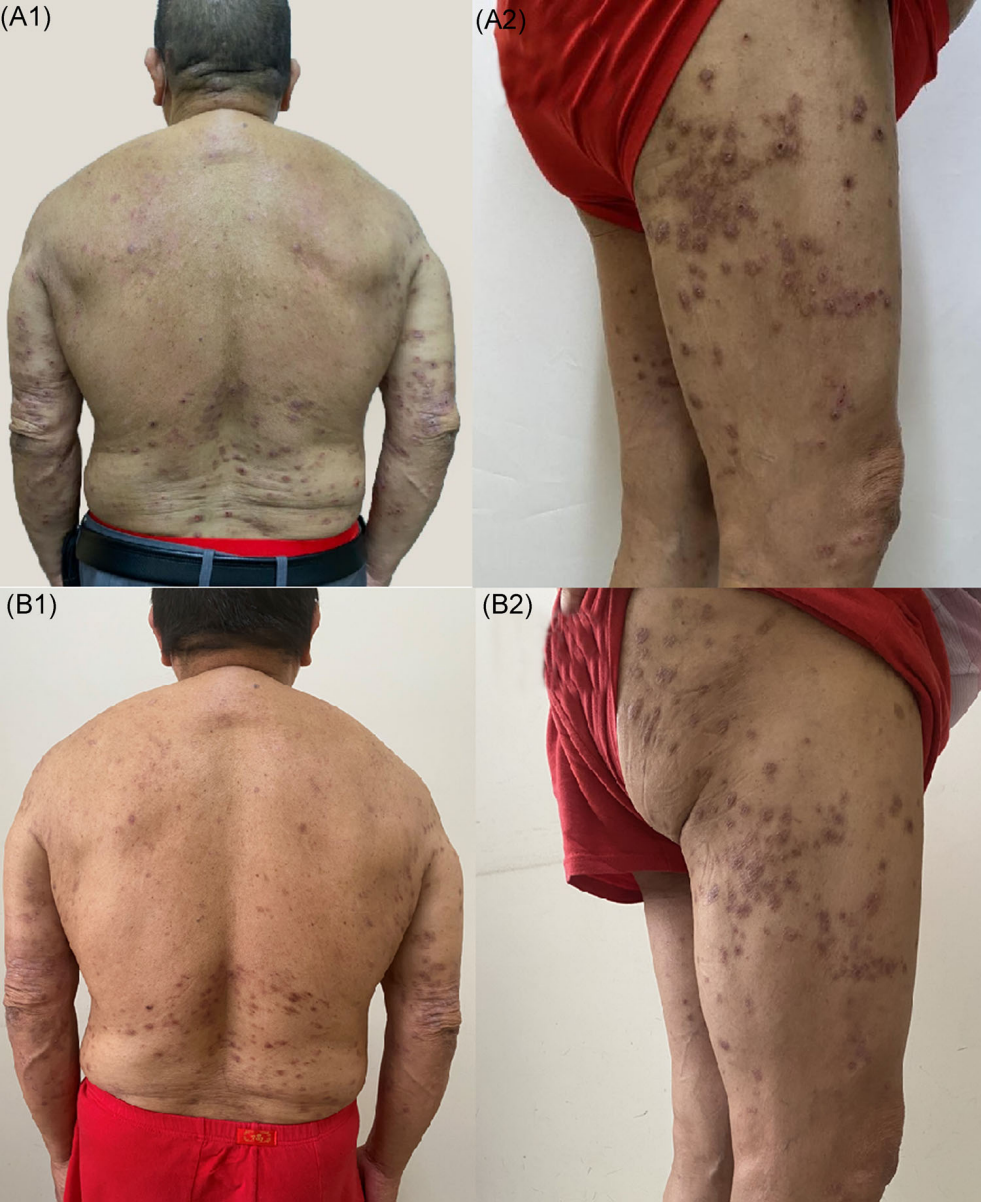
Clinical picture of Patient 2 before (a) and after (b) 6 weeks of treatment with dupilumab

**Figure 4:**
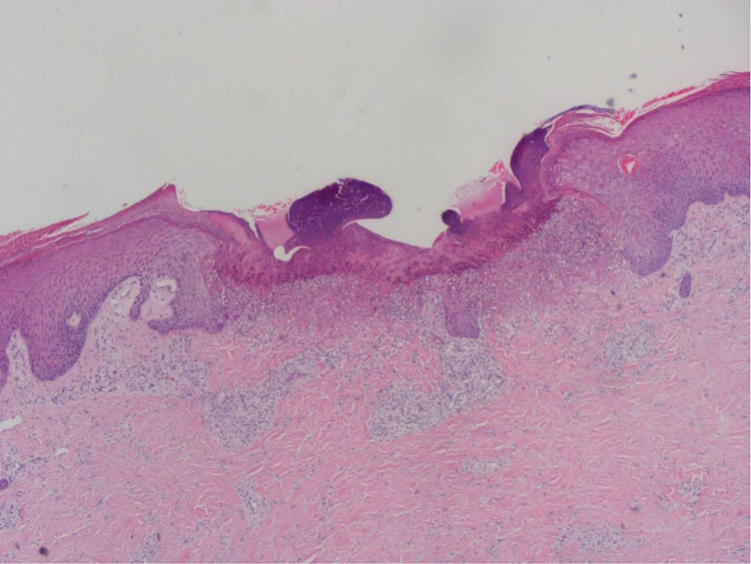
Histopathological picture for Patient 2

A 70‐year‐old man presented with multiple pruritus papules and nodules on his trunk and limbs for 2 years (Figure 3A1,A2). He has a 10 years history of well‐controlled type 2 diabetes. He was treated with oral antihistamine and topical potent steroids without any improvement for several months. Dermatological examination revealed generalized scratches and umbilicated papules or nodules with symmetric distribution on his trunk and extremities. Histopathological examination for umbilicated papule suggested ARPC (Figure 4). Laboratory examination revealed the increased eosinophils count (1.74 × 10^9^/L) and normal total IgE (27.62IU/ml). He was diagnosed with ARPC and elderly AD. After obtaining informed consent, we initiated dupilumab monotherapy at a dosage of 600 mg, followed by 300 mg twice a month. Six weeks after treatment, his eruptions and itching improved substantially (Figure 3B1,B2). During follow‐up, the patient continued to use dupilumab without recurrence and any adverse effects.

## 3 DISCUSSION

AD is a chronic recurrent eczema dermatitis that usually affects young patients, recent reports highlighted the appearance of the disease in the elderly with a slight prevalence for male patients.^3^ Both two old males in this report are diagnosed as elderly AD based on the presence of an eczematous or pruriginous pattern of dermatitis, itchiness, and relapsing course. There are no specific guidelines for doctors to distinguish AD from other itchy skin conditions in the elderly. Currently, elderly patients diagnosed with AD require at least 6 months for symptom evaluation and exclusion of others including cutaneous T‐cell lymphoma, allergic contact dermatitis, drug reactions, and chronic idiopathic or secondary erythroderma.^4^

ARPC is a rare form of trans epithelial elimination in which altered collagen is extruded through the epidermis. Gambichler et al.^5^ suggested that the transepithelial elimination of collagen is just a response mode related to chronic scratching. When the pruritus is controlled, the lesions of ARPC usually resolve and the skin lesions will disappear within a few months.^6^

More recently, IL‐4 and IL‐13 have been shown to act directly on itching neurons to promote itching, and IL‐4Rα, a part of IL‐4 receptor and IL‐13 receptor, has been found to be expressed and functionally active on mouse and human sensory neurons.^7^ Dupilumab, a fully human monoclonal antibody against IL‐4Rα, has been approved for treating moderate‐to‐severe AD^8^ and has also been proved effective in patients with prurigo nodularis, pruritic lichenified nodules, lichen planus, and other pruritic disorders such as chronic pruritus of unknown origin, uremic pruritus, and malignancy‐associated pruritus.^7^ Our patient was treated with dupilumab to relieve the itching, and the rash subsided after the itching stopped.

In these two cases, the utilization of dupilumab to treat ARPC combined with elderly AD has shown a satisfying efficacy. Our successful experience in ARPC suggests that dupilumab may serve as an alternative option in treating ARPC combined with elderly AD.

## CONFLICT OF INTERESTS

The authors declare that there are no conflict of interests.
